# Intracerebroventricular administration of oxytocin and intranasal administration of the oxytocin derivative improve β‐amyloid peptide (25–35)‐induced memory impairment in mice

**DOI:** 10.1002/npr2.12292

**Published:** 2022-09-19

**Authors:** Junpei Takahashi, Yudai Ueta, Daisuke Yamada, Sachie Sasaki‐Hamada, Takashi Iwai, Tomomi Akita, Chikamasa Yamashita, Akiyoshi Saitoh, Jun‐Ichiro Oka

**Affiliations:** ^1^ Laboratory of Pharmacology, Faculty of Pharmaceutical Sciences Tokyo University of Science Chiba Japan; ^2^ Department of Physiology, School of Allied Health Sciences Kitasato University Sagamihara Japan; ^3^ Laboratory of Pharmacology, School of Pharmaceutical Sciences Kitasato University Tokyo Japan; ^4^ Laboratory of Pharmaceutics and Drug Delivery, Faculty of Pharmaceutical Sciences Tokyo University of Science Chiba Japan

**Keywords:** amnesia, brain drug delivery and targeting, intranasal administration, oxytocin, spatial memory, β‐amyloid peptide (25–35)

## Abstract

**Aim:**

We previously reported that oxytocin, a peptide hormone, can reverse the β‐amyloid peptide (25–35) (Aβ_25–35_)‐induced impairments of the murine hippocampal synaptic plasticity. In this study, we examined the effects of oxytocin on the Aβ_25–35_‐induced impairment of cognitive behavior in murine in order to investigate the potential of oxytocin as a clinical treatment tool for Alzheimer's disease (AD).

**Methods:**

The Y‐maze and Morris water maze (MWM) tests were performed. Since the intracerebroventricular (ICV) administration is both invasive and impractical, we further utilized intranasal (IN) delivery to the brain. For this purpose, we prepared an oxytocin derivative containing cell‐penetrating peptides and a penetration accelerating sequence, which was subsequently used in our IN administration experiments.

**Results:**

We herein showed that the ICV administration of oxytocin in mice exerted memory‐improving effects on the Aβ_25–35_‐induced amnesia in both the Y‐maze and MWM tests. The IN administration of the oxytocin derivative exhibited memory‐improving effects in the Y‐maze test. Moreover, we acquired evidence that the fluorescein isothiocyanate‐labeled oxytocin derivative was distributed throughout the mouse brain following its IN administration.

**Conclusion:**

Our results suggest that the oxytocin derivative is effective for its IN delivery to the brain and may be particularly useful in the clinical treatment of cognitive impairment, such as that characterizing AD.

## 1 INTRODUCTION

Alzheimer's disease (AD) is the most common cause of dementia in the elderly, characterized by progressive memory loss and β‐amyloid protein (Aβ) accumulation in the brain. Aβ consists of 39‐43 amino acids, and the intracerebroventricular (ICV) administration of Aβ generates certain murine AD models. Previous studies showed that the ICV injection of Aβ impaired cognitive behavior and synaptic plasticity in mice.^1^, ^2^, ^3^, ^4^

Oxytocin, a peptide hormone synthesized in the paraventricular hypothalamic nucleus (PVH) and the supraoptic nucleus, facilitates parturition and lactation. In the rodent central nervous system (CNS), oxytocin regulates social behavior, anxiety, depression and cognitive behavior.^5^ The oxytocin receptors and oxytocinergic neurons are identifiable in various CNS areas.^6^, ^7^, ^8^, ^9^ We recently reported that oxytocin could reverse the amyloid β_25–35_ peptide (Aβ_25‐35_)‐induced impairments of hippocampal synaptic plasticity in mice^10^ since oxytocin perfusion showed recovery from the Aβ_25–35_‐induced impairments of long‐term hippocampal potentiation (LTP) via the oxytocin receptor. Furthermore, in that same study, we identified that the extracellular signal‐regulated kinase (ERK) and the Ca^2+^‐permeable α‐amino‐3‐hydroxy‐5‐methyl‐4‐isoxazolepropionic acid (AMPA) receptor are involved in this effect.^10^ Actually, a clinical study reported that AD patients had a lower right hippocampus volume and plasma oxytocin concentration than the control group.^11^ Based on these results, we proposed that oxytocin should be considered a novel treatment for memory loss associated with cognitive disorders, such as AD. However, there has been little research on whether the administration of oxytocin could reverse the Aβ‐induced impairments of cognitive behavior through in vivo studies. Therefore, the present study aimed to examine whether oxytocin could reverse the Aβ_25–35_‐induced impairments of spatial memory.

In general, peptides are characterized by poor blood‐brain barrier (BBB) permeability.^12^ The ICV administration of peptides, usually used in animal studies, is very invasive and impractical for a potential clinical application. Intranasal (IN) administration is reportedly a clinically applicable technique for the delivery of proteins and peptides into the CNS.^13^ We have already developed a new technique for the efficient delivery of peptide derivatives containing cell‐penetrating peptides (CPPs) and a penetration accelerating sequence (PAS) to the brain through an IN administration (European patent: EP‐3‐190‐129‐B1; January 8, 2020, JPN patent pending: No. 2014‐184 436, International publication number: WO 2016/035820; March 10, 2016), and have reported that the IN administration of a glucagon‐like peptide‐2 (GLP‐2) derivative and of a neuromedin U derivative exert antidepressant‐like and memory‐improving effects, respectively, in mice.^14^, ^15^ CPPs interact with the proteoglycan layer of the cellular membrane^16^, ^17^, ^18^, ^19^ and can be delivered into the cell by macropinocytosis.^20^, ^21^ Moreover, PAS reportedly promotes the escape from the endosomal membrane.^22^ A previous study demonstrated the usefulness of both PAS and CPPs for nose‐to‐brain delivery.^15^, ^23^

In the present study, we applied our technique to oxytocin. We prepared an oxytocin derivative containing PAS and CPPs (PAS‐CPPs‐oxytocin) to examine whether the IN administration of this oxytocin derivative or the ICV administration of native oxytocin can improve the Aβ_25–35_‐induced impairments of learning and memory behavior in mice.

## 2 METHODS

### 2.1 Animals

The Institutional Animal Care and Use Committee at Tokyo University of Science approved all animal study protocols. The experiments complied with the National Institute of Health and the Japan Neuroscience Society guidelines. We used 6‐ to 7‐week‐old male ddY mice (Japan SLC, Inc., Shizuoka, Japan). All animals had free access to food and water and were housed in an animal facility room with maintained temperature (23 ± 1°C) and relative humidity (55 ± 5%), and with a 12‐hours light‐dark cycle (lights switched on automatically at 8:00 am).

### 2.2 Drug administration

The ICV administration (5 μL/ventricle) was performed according to the previously reported procedures^24^ using a 50‐μL Hamilton microsyringe with a 28‐gauge needle (KN‐386; Natsume Seisakusho Co, Ltd, Tokyo, Japan) under brief isoflurane anesthesia. The IN administration (2 μL per each nostril) was performed according to the previously reported procedures^14^, ^15^ using micropipette in both nostrils under brief isoflurane anesthesia. Nose drops were administered to animals lying on their backs for consistent deposition in the olfactory or respiratory epithelium.

### 2.3 Amyloid β_25–35_ peptide‐induced amnesia model (Aβ model)

Aβ_25–35_ from Peptide Inc. (Osaka, Japan) was prepared as described by Maurice et al^25^, ^26^ with some modifications. Aβ_25–35_ was dissolved in 5% dimethyl sulfoxide (DMSO; Wako Pure Chemical Industries, Osaka, Japan) at a concentration of 1.74 mg/mL, and then AlCl_3_ · 6H_2_O (3.4 mg/mL; Wako Pure Chemical Industries, Osaka, Japan) was added. Before injection, Aβ_25–35_ was aggregated by incubation at 37°C for 4 days. Aβ model was established by ICV administration of the Aβ_25–35_ solution.

### 2.4 Drug

Oxytocin (human: MW = 1007.2) was obtained from Peptide, Inc. (Osaka, Japan), while the oxytocin receptor antagonist, L‐368899 came from R&D Systems (Minneapolis, MN, USA). All drugs were dissolved in 0.9% saline (vehicle). The oxytocin derivative containing PAS and CPPs (FFLIPKG‐RRRRRRRR‐GG‐oxytocin‐NH_2_) labeled with fluorescein isothiocyanate (FITC) was synthesized by the SCRUM, Inc. (Tokyo, Japan) with a peptide synthesizer (433A; Applied Biosystems) following a standard 9‐fluorenylmethoxycarbonyl method. The FITC‐labeled oxytocin derivative (MW = 3676.3) was dissolved in 0.9% saline (1 μg/4 μL).

### 2.5 Y‐maze test

We examined spatial working memory by measuring the spontaneous alternation behavior of mice in the Y‐maze test, as described previously with some modifications.^3^, ^14^ The maze was made of black acrylic board. Each arm was 40 cm long, 12 cm high, 3 cm wide at the bottom, 10 cm wide at the top, and converged at an equal angle. Each mouse was at the end of one arm and allowed to move freely through the maze during an 8‐minutes session. The series of arm entries were recorded visually. Alternation was defined as successive entries into the three arms on overlapping triplet sets. The effect was calculated as the percent alternation by using the following formula: alternation (%) = {(number of alternations)/(total number of arm entries − 2)} × 100 (%). The maze arms were wiped down with paper between sessions.

### 2.6 Morris water maze test

We examined spatial reference memory by measuring the latency to target in the Morris water maze (MWM) test as previously described, with some modifications.^27^ A circular pool made of gray polyvinylchloride (100 cm in diameter and 50 cm high) was filled to a depth of 25 cm with clear water (25 ± 1°C) and surrounded by a black curtain with distant spatial markers. There was an escape platform (target) submerged 1 cm under the water that was 15 cm in diameter. Each mouse underwent four daily 120 seconds trials for three consecutive days with intertrial intervals between 30 minutes. For each training trial, mice were released into the water facing the pool wall from semirandomly chosen cardinal compass points (north, east, south, and west). On the fourth day, four sessions of the 2‐minutes probe trials were performed to determine whether the mice understood the platform location, and we measured the time within the target zone without an escape platform. All trials were recorded and analyzed by spontaneous motor activity recording using computer software (SMART3; Panlab SL, Barcelona, Spain).

### 2.7 Evaluation of the locomotor activity

A multichannel activity‐counting system and an open‐field apparatus evaluated locomotor activity. The multichannel activity‐counting system (Supermex) instrument (Muromachi Kikai, Tokyo, Japan) can monitor even minute movements. Its infrared sensor with multiple Fresnel lenses (that can be moved close enough to the cage) can capture multidirectional locomotor alternations in a single mouse. The Supermex instrument was connected to a behavior‐analyzing system (CompACT AMS, Muromachi Kikai, Tokyo, Japan) that can interpret each movement as one count. The open‐field apparatus consisted of a square area (40 × 40 cm) with 25‐cm‐high black acrylic walls, as described previously with some modifications.^15^ Lines were drawn and were used to divide the open‐field apparatus into 16 fractions; as a result, the spontaneous activity was counted manually as the number of line crossings that occurred. In each experiment, mice were allowed to move freely during a 5‐minutes session.

### 2.8 Distribution of the intranasally administrated oxytocin derivative in the mouse brain

Mice were perfused transcardially with 0.1 M of phosphate buffer (PB, pH 7.4), followed by 50‐100 mL of 4% (w/v) paraformaldehyde. Their brains were removed and postfixed at 4°C overnight in the same fixative. After cryoprotection with 30% (w/v) sucrose in phosphate‐buffered saline, the brains were sectioned by a cryostat (CM1560S; Leica Microsystems, Wetzlar, Germany) at 30 μm, into five series. We washed the tissue sections in PB twice and analyzed their fluorescence patterns microscopically (BZ‐9000; Keyence, Osaka, Japan). We counted FITC‐positive dots per tissue section using Dynamic cell count BZ‐HIC (Keyence) software. To ascertain brain regions, we stained alternate sections with 0.2% cresyl violet for Nissl substance in the following areas according to Paxinos and Franklin^28^: olfactory bulb (OB; bregma 3.92 mm), infralimbic cortex (IL; bregma 1.54 mm), PVH (bregma −0.70 to −0.94 mm), basolateral amygdala (BLA; bregma −1.34 to −1.82), hippocampus (HIP; bregma −1.34 to −1.82 mm), dorsomedial hypothalamic nucleus (DMH; bregma −1.46 mm), principal sensory trigeminal nucleus (Pr5; bregma −5.33 mm), and rostral ventrolateral medulla (RVL; bregma −6.59 mm).

### 2.9 Statistical analysis

Data are represented as mean ± standard error of the mean. We evaluated significant differences in the Y‐maze test, the probe trial of the MWM test, and locomotor activity data using a one‐way analysis of variance (ANOVA) followed by Bonferroni's multiple comparison test. For day 1‐3 trials of the MWM test data, we evaluated significant differences using a two‐way no matching ANOVA followed by Bonferroni's post hoc test. We evaluated significant differences in relative fluorescence area data using Mann‐Whitney's *U* test. Analyses were performed using Graphpad Prism (Graphpad Software, Inc., San Diego, CA, USA); a *P‐*value of <0.05 was considered statistically significant.

## 3 RESULTS

### 3.1 Effects of oxytocin (ICV) on Aβ‐induced murine amnesia in the Y‐maze test

**FIGURE 1:**
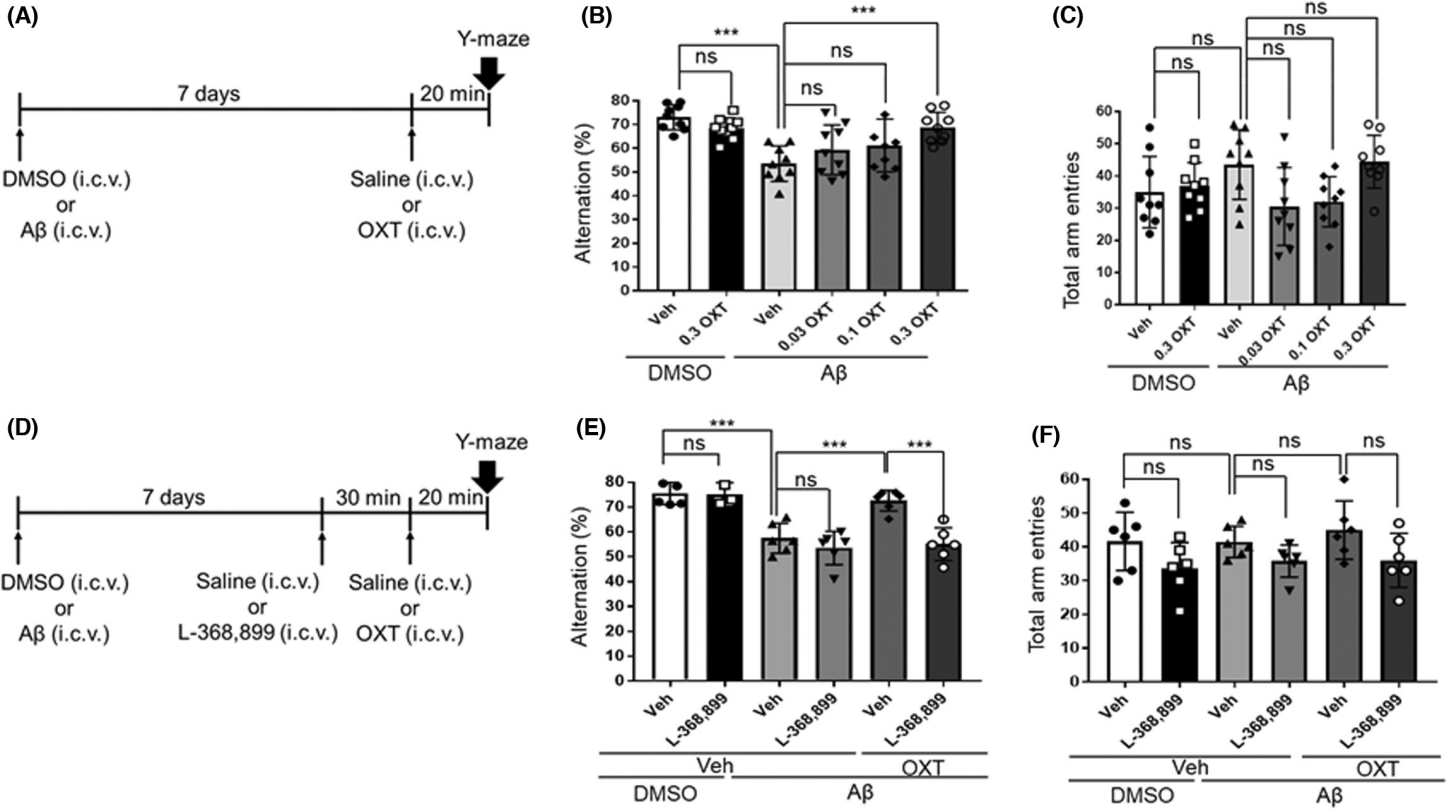
The effects of oxytocin on murine Aβ‐induced amnesia in the Y‐maze test are mediated via the oxytocin receptor. Mice received an ICV administration of Aβ (8.2 nmol) or 5% DMSO. An overview of the experimental schedule is presented in A. The Y‐maze test was performed on day 7. Effects of oxytocin on the murine Aβ‐induced amnesia in the Y‐maze test: B, percent alteration [*F*(5,48) = 7.519, *P* < 0.0001]; C, total arm entries during an 8‐minutes session [*F*(5,48) = 3.219, *P* = 0.0138]. An overview of the experimental schedule is present in D.Effects of the oxytocin receptor antagonist (L‐368899) on Y‐maze test performance: E, percent alteration [*F*(5,30) = 21.76, *P* < 0.0001]; F, total arm entries during 8‐minutes session [*F*(5,30) = 2.21, *P* = 0.0794]. Vehicle solution (saline, Veh) or L‐368899 (2 μg) were intracerebroventricularly administered 50 minutes before the test, and vehicle solution (saline, Veh) or oxytocin (0.03‐0.3 μg) was intracerebroventricularly administered 20 minutes prior to the test. The number of animals per group was n = 9. Data are presented as mean ± SEM. ***P < 0.001, ns: not significant (one‐way ANOVA followed by Bonferroni's multiple comparison test). ANOVA, analysis of variance; DMSO, dimethyl sulfoxide; ICV, intraventricular; SEM, standard error of the mean

We examined whether an ICV oxytocin administration could improve the spatial working memory impairment induced by Aβ on mice. Figure 1A presents the experimental schedule. The ICV administration of oxytocin (0.3 μg/5 μL) significantly improved the impairment of spontaneous alternation performance induced by Aβ (Figure 1B) [*F*(5,48) = 7.519, *P* < 0.0001, one‐way ANOVA; *P* < 0.001 for Aβ‐vehicle vs Aβ‐0.3 μg oxytocin, Bonferroni's post hoc test]. Oxytocin did not affect the spontaneous alternation performance of DMSO‐treated control mice (Figure 1B). Moreover, oxytocin and Aβ did not affect the total arm entries (Figure 1C) [*F*(5,48) = 3.219, *P* = 0.0138, one‐way ANOVA; ns, *P* > 0.05 for Aβ‐vehicle vs Aβ‐0.3 μg oxytocin, Bonferroni's post hoc test].

### 3.2 The inhibition of the beneficial effects of oxytocin (ICV) on Aβ‐induced murine amnesia on the Y‐maze test by a prior administration of the oxytocin receptor antagonist, L‐368899

We examined whether the beneficial effects of oxytocin, as displayed in the Y‐maze test, were mediated by oxytocin receptors (Figure 1D). Prior administration of the oxytocin receptor antagonist, L‐368899 (2 μg/5 μL, ICV), significantly inhibited the improving effects of oxytocin on the impairment of spontaneous alternation performance induced by Aβ (Figure 1E) [*F*(5,30) = 21.76, *P* < 0.0001, one‐way ANOVA; *P* < 0.001 for Aβ‐vehicle‐oxytocin vs Aβ‐L‐368899‐oxytocin, Bonferroni's post hoc test]. However, the administration of L‐368899 on its own exerted no effects on the spontaneous alternation performance of either the DMSO or the Aβ‐treated mice (Figure 1E). The administration of L‐368899 did not affect the total arm entries either (Figure 1F) [*F*(5,30) = 2.21, *P* = 0.0794, one‐way ANOVA; ns, *P* > 0.05 for Aβ‐vehicle vs Aβ‐0.3 μg oxytocin, Bonferroni's post hoc test].

### 3.3 Effects of oxytocin (ICV) on Aβ‐induced murine amnesia in the MWM test

**FIGURE 2:**
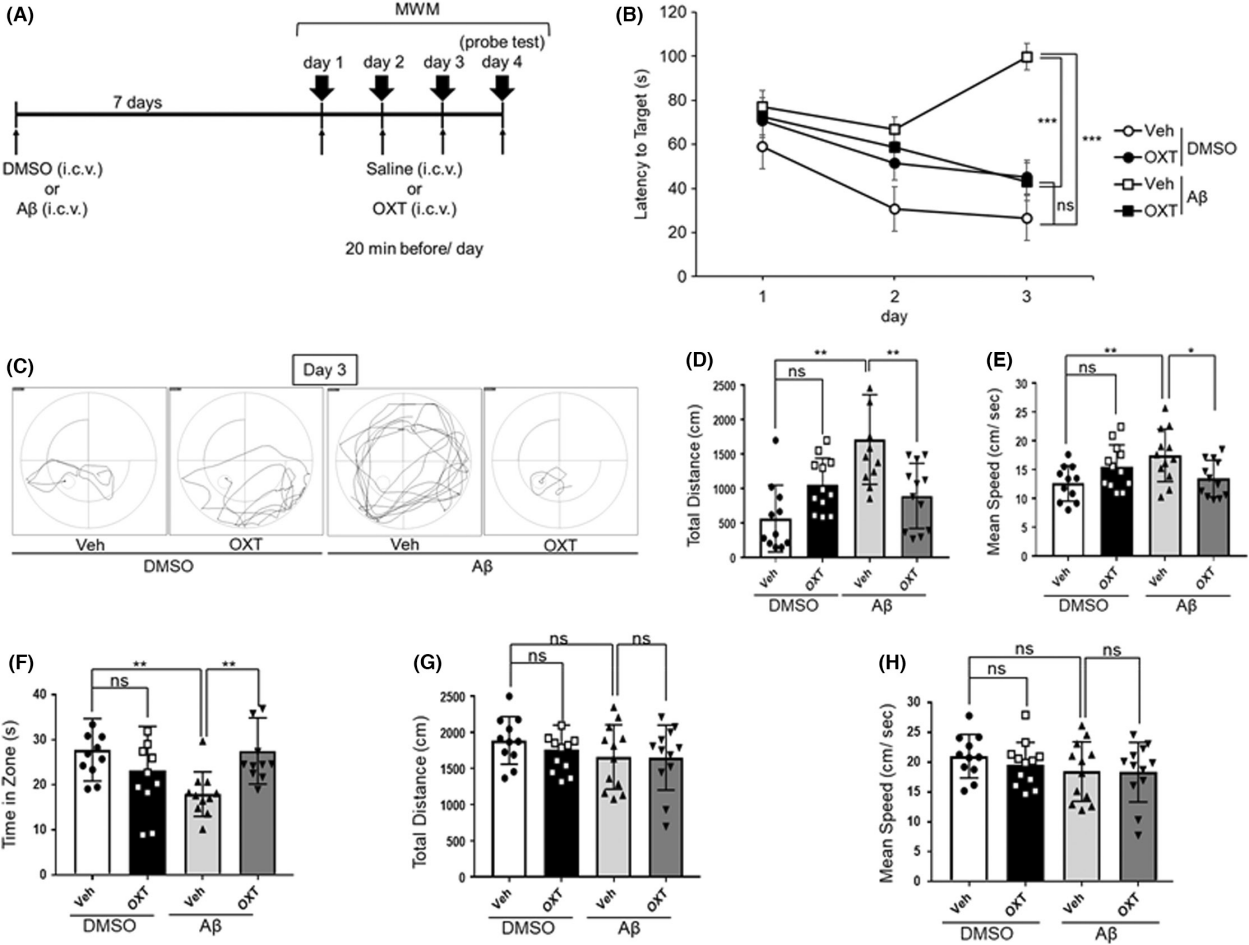
Effects of oxytocin on murine Aβ‐induced amnesia in the Morris water maze (MWM) test. Mice received an ICV administration of Aβ (8.2 nmol) or 5% DMSO. An overview of the experimental schedule is presented in A. The MWM hidden platform test was performed on days 8‐10: B, latency to target [drugs; *F*(3,129) = 16.82, *P* < 0.0001, trial days; *F*(2,129) = 3.77, *P* = 0.00017, interaction between drugs and trial days; *F*(6,129) = 7.36, *P* = 0.0009]; C, representative traces of swimming; D, total distance of swimming [*F*(3,43) = 10.49, *P* < 0.0001]; E, mean swimming speed during four 2‐minutes trials per day, for 3 days [*F*(3,43) = 3.98, *P* = 0.0136]. The MWM probe test was performed on day 11: F, time spent within the target zone [*F*(3,43) = 4.891, *P* = 0.0052]; G, total distance of swimming [*F*(3,43) = 0.9047, *P* = 0.4467]; H, mean swimming speed during four 2‐minutes trials [*F*(3,43) = 0.905, *P* = 0.4466]. Vehicle solution (saline, Veh) or oxytocin (0.3 μg) were intracerebroventricularly administered 20 minutes before the test. The number of animals per group was n = 11‐12. Data are presented as mean ± SEM. *P < 0.05, **P < 0.01, ***P < 0.001, ns: not significant [two‐way no matching ANOVA followed by Bonferroni's post hoc test (B), and one‐way ANOVA followed by Bonferroni's multiple comparison test (D–H)]. ANOVA, analysis of variance; DMSO, dimethyl sulfoxide; ICV, intraventricular; SEM, standard error of the mean

We examined whether an ICV oxytocin administration could improve the spatial reference memory impairment induced by Aβ on mice. Figure 2A presents the experimental schedule. ICV administration of oxytocin (0.3 μg) 20 minutes before every trial reduced the latency to target (extended by Aβ) on day 3 (Figure 2B) [drugs; *F*(3,86) = 15.28, *P* < 0.0001, trial days; *F*(2,86) = 7.753, *P* = 0.0008, interaction between drugs and trial days; *F*(6,86) = 3.970, *P* = 0.0015, two‐way repeated‐measured ANOVA; *P* < 0.001 for Aβ‐vehicle vs Aβ‐oxytocin on day 3, Bonferroni's post hoc test]. Figure 2C provides representative swimming orbits on day 3. The ICV administration of Aβ significantly extended the recorded total distance (Figure 2D) and increased the recorded mean speed (Figure 2E). Moreover, ICV administration of oxytocin extended the time spent in the target zone, which was reduced by Aβ in the probe trial on day 4 (Figure 2F) [*F*(3,43) = 4.891, *P* = 0.0052, one‐way ANOVA; *P* < 0.01 for Aβ‐vehicle vs Aβ‐oxytocin on day 4, Bonferroni's post hoc test]. ICV administration of Aβ or oxytocin did not affect the recorded total distance (Figure 2G) [*F*(3,43) = 0.9047, *P* = 0.4467, one‐way ANOVA; ns, *P* > 0.05 for Aβ‐vehicle vs Aβ‐oxytocin on day 4, Bonferroni's post hoc test] and the mean speed (Figure 2H) [*F*(3,43) = 0.905, *P* = 0.4466, one‐way ANOVA; ns, *P* > 0.05 for Aβ‐vehicle vs Aβ‐oxytocin on day 4, Bonferroni's post hoc test].

### 3.4 Effect of native oxytocin (IN) and of the oxytocin derivative (IN) on Aβ_25–35_‐induced murine amnesia in the Y‐maze test

**FIGURE 3:**
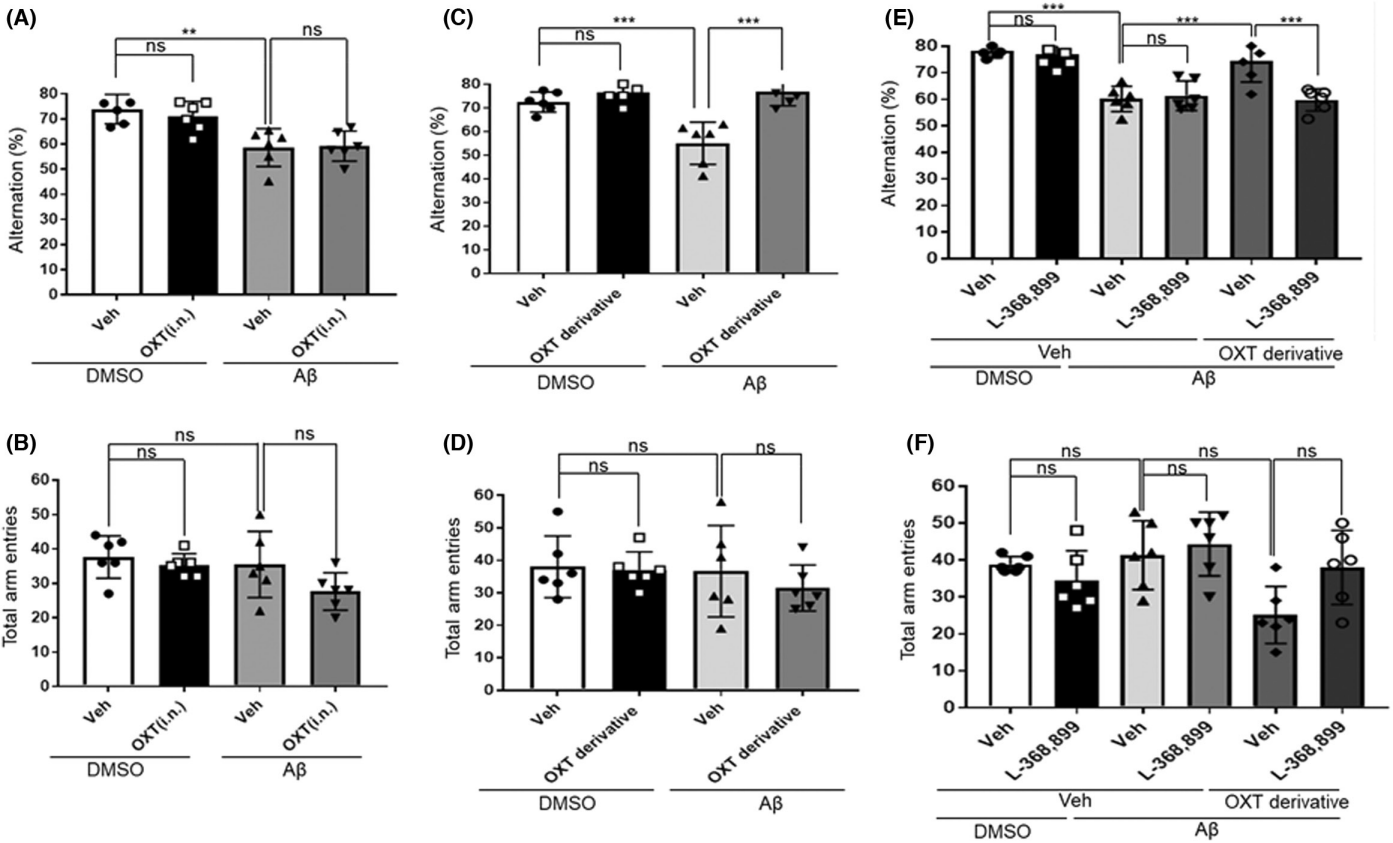
Pretreatment with the oxytocin receptor antagonist (L‐368899) inhibits the beneficial effects of the oxytocin derivative on the murine Aβ‐induced amnesia in the Y‐maze test. Mice received an ICV administration of Aβ (8.2 nmol) or 5% DMSO. The Y‐maze test was performed on day 7. Effect of IN administration of the native oxytocin on the Y‐maze test performance of mice: A, percent alteration [*F*(3,20) = 9.209, *P* = 0.0005]; B, total arm entries during an 8‐minutes session [*F*(3,20) = 2.698, *P* = 0.0732]. Effect of the IN oxytocin derivative on the Y‐maze test performance of mice: C, percent alteration [*F*(3,20) = 16.91, *P <* 0.0001]; D, total arm entries during an 8‐minutes session [*F*(3,20) = 0.5527, *P* = 0.6522]. Effect of the ICV administration of the oxytocin receptor antagonist (L‐368899) on the Y‐maze test performance of mice: E, percent alternation [*F*(5,30) = 16.28, *P <* 0.0001]; F, total arm entries during an 8‐minutes session [*F*(5,30) = 4.121, *P* = 0.0057]. Vehicle solution (saline, Veh) or L‐368899 (2 μg) were intracerebroventricularly administered 50 minutes before the test, and the vehicle solution (saline, Veh), the native oxytocin (0.6 μg) or the oxytocin derivative (2 μg) was intranasally administered 20 minutes before the test. The number of animals per group was n = 6. Data are presented as mean ± SEM. **P < 0.01, ***P < 0.001, ns: not significant (one‐way ANOVA followed by Bonferroni's multiple comparison test). ANOVA, analysis of variance; DMSO, dimethyl sulfoxide; ICV, intraventricular; IN, intranasal; SEM, standard error of the mean

We examined whether IN administration of oxytocin affects the Aβ_25–35_‐induced impairment of spatial working memory in mice. The IN administration of native oxytocin (0.3 μg/4 μL) failed to recover the Aβ_25–35_‐induced impairment of spontaneous alternation (Figure 3A) [*F*(3,20) = 9.209, *P* = 0.0005, one‐way ANOVA; ns, *P >* 0.05 for Aβ‐vehicle vs Aβ‐0.3 μg native oxytocin, Bonferroni's post hoc test]. The IN administration of the oxytocin derivative (1 μg/4 μL) reversed the Aβ_25–35_‐induced impairment of spontaneous alternation (Figure 3C) [*F*(3,20) = 16.91, *P <* 0.0001, one‐way ANOVA; *P <* 0.0001 for Aβ‐vehicle vs Aβ‐1.0 μg oxytocin derivative, Bonferroni's post hoc test]. Moreover, the prior administration of L‐368899 (2 μg, ICV) significantly inhibited the beneficial effects of the oxytocin derivative on the impairment of spontaneous alternation performance induced by Aβ_25–35_ (Figure 3E) [*F*(5,30) = 16.28, *P <* 0.0001, one‐way ANOVA; *P <* 0.0001 for Aβ‐vehicle vs Aβ‐L‐368899—2.0 μg oxytocin derivative, Bonferroni's post hoc test]. None of the treatments affected the recorded total arm entries (Figure 3B,D,F).

### 3.5 Distribution of the FITC‐labeled oxytocin derivative in the murine brain

**FIGURE 4:**
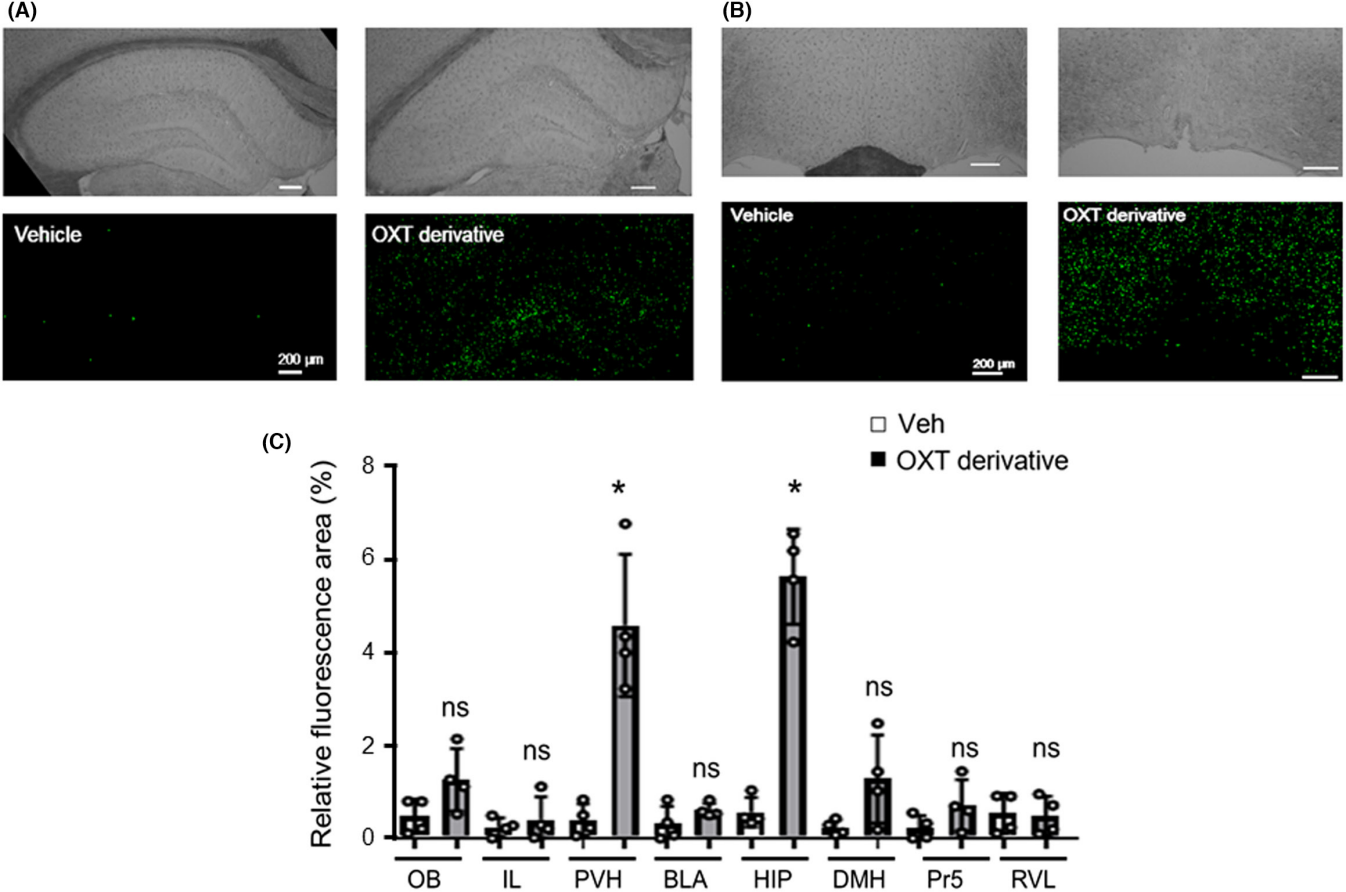
Regional brain distribution of the FITC‐labeled oxytocin derivative in murine brain tissue 20 minutes after its intranasal administration. Mice received an IN administration of the vehicle solution (saline, Veh) or the FITC‐labeled oxytocin derivative (2 μg). Fluorescence microscopy observation of cryosections from murine brains obtained 20 minutes after the IN administration: A, fluorescence microscopy images of the hippocampus (HIP). *Fluorescence signals*: green. B, Fluorescence microscopy images of the paraventricular hypothalamic nucleus (PVH) *Fluorescence signals*: green. C, Relative fluorescence area percentage in each brain region (Mann‐Whitney *U* = 0.0000 for PVH and HIP). DMH, dorsomedial hypothalamic nucleus; IL, infralimbic cortex; OB, olfactory bulb; Pr5, principal trigeminal nucleus; RVL, rostral ventrolateral pressor area. The number of cryosections per group was n = 4. Data are presented as mean ± SEM. *P < 0.05, ns: not significant (Mann‐Whitney's *U* test). FITC, fluorescein isothiocyanate; IN, intranasal

We investigated the distribution of the FITC‐labeled oxytocin derivative after IN administration. There were fluorescence signals in the PVH and the HIP 20 minutes after the IN administration (Figure 4A,B). We analyzed the relative fluorescence area (%) in various brain regions (for more details, see Section 2.8). The relative fluorescence area (%) increased significantly in the PVH and the HIP 20 minutes after the IN administration (*P* < 0.05, Mann‐Whitney's *U* test), but not in other brain regions (ns, *P* > 0.05, Mann‐Whitney's *U* test) (Figure 4C).

### 3.6 Influence of oxytocin and Aβ on murine locomotor activity

**FIGURE 5:**
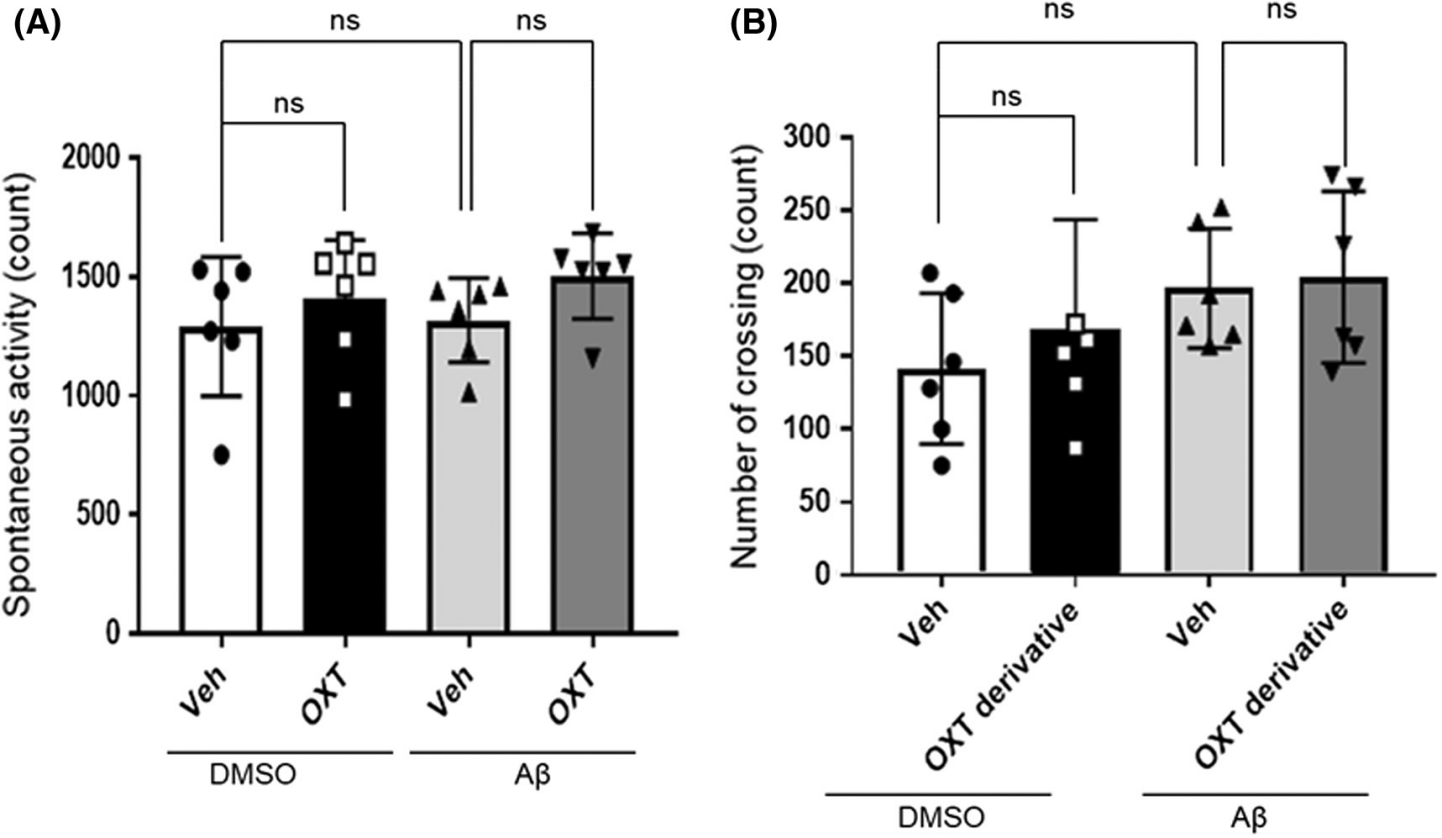
Influences of oxytocin and Aβ on the spontaneous activity of mice. A, Spontaneous activity recording during a 5‐minutes session. Vehicle solution (saline, Veh) or oxytocin (0.3 μg) was intracerebroventricularly administered 20 minutes before the recording. The number of animals per group was n = 6. Data are presented as mean ± SEM. ns: not significant. [*F*(3,20) = 1.034, *P* = 0.3987] (one‐way ANOVA followed by Bonferroni's multiple comparison test). B, The open‐field test was performed on day 7: the number of crossing lines during a 5‐minutes session. Vehicle solution (saline, Veh) or the oxytocin derivative (2 μg) was intranasally administered 20 minutes before the test. The number of animals per group was n = 6. Data are presented as mean ± SEM. ns: not significant. [*F*(3,20) = 1.457, *P* = 0.2562] (one‐way ANOVA followed by Bonferroni's multiple comparison test). ANOVA, analysis of variance; SEM, standard error of the mean

Since murine spatial memory is often associated with spontaneous activity, we examined whether the native oxytocin could influence spontaneous locomotor activity in the Aβ_25–35_‐treated mice. The ICV administration of oxytocin did not affect the locomotor activity (counts) in either the DMSO‐ or the Aβ_25–35_‐treated mice [*F*(3,20) = 1.034, *P* = 0.3987, one‐way ANOVA; ns, *P* > 0.05 for DMSO‐vehicle vs Aβ‐vehicle or Aβ‐vehicle vs Aβ‐oxytocin, Bonferroni's post hoc test] (Figure 5A). Subsequently, we examined whether the oxytocin derivative (IN) or the Aβ_25–35_ (ICV) could affect the locomotor activity in the open‐field test. There were no significant differences identified in the number of crossing lines between the oxytocin derivative (IN) and/or the Aβ_25–35_ (ICV) treatment groups compared with their respective vehicle (control) treatment groups (Figure 5B) [*F*(3,20) = 1.457, *P* = 0.2562, one‐way ANOVA; ns, *P* > 0.05 for DMSO‐vehicle vs Aβ‐vehicle or Aβ‐vehicle vs Aβ‐oxytocin derivative, Bonferroni's post hoc test].

## 4 DISCUSSION

For the first time, this study demonstrates that ICV administration of native oxytocin and IN administration of the oxytocin derivative can recover the Aβ_25–35_‐induced impairments of memory in mice without affecting their locomotor activities. Oxytocin receptors mediate the memory‐improving effects of native oxytocin (ICV) and the oxytocin derivative (IN). As shown in Figure 1B,C, the ICV of native oxytocin was significantly effective at a dose of 0.3 μg in dose‐response examination of three doses, and this dose was used thereafter (Figures 1E,F, 2 and 3A,B). Also, the amounts of injected oxytocin were similar in mole number, 0.298 nmol for 0.3 μg of native oxytocin and 0.272 nmol for 1.0 μg of the oxytocin derivative. Our previous reports have shown that IN of the derivatives of GLP‐2 and neuromedin U induced the similar pharmacological effects at equivalent or smaller amounts compared to ICV of those native peptides; a fact suggestive of a very effective nose‐to‐brain delivery.^14^, ^15^, ^23^ The purpose of this study was to confirm whether the IN of oxytocin derivative would have a pharmacological effect equivalent to the ICV of native oxytocin, so we tested it at one dose that would be expected to be potent. Furthermore, fluorescence imaging demonstrated that the oxytocin derivative's delivery to the brain, including the HIP, via the IN administration route. Based on these findings, we believe oxytocin provides a protective effect against the memory impairments linked to pathological conditions such as AD, rather than a strengthening effect on memory under normal (healthy) conditions. The oxytocin derivative could probably prove useful in the clinical treatment of AD.

We recently showed that the perfusion of oxytocin can recover the Aβ_25–35_‐induced impairments of hippocampal LTP via the oxytocin receptors.^10^ This finding strongly supports our suggestion that oxytocin could be effective in the treatment of Aβ‐induced impairments of cognitive behavior in mice. Moreover, we have previously suggested that the ERK phosphorylation and the Ca^2+^‐permeable AMPA receptors could be involved in the beneficial effects of oxytocin on the Aβ_25–35_‐induced impairments of synaptic plasticity in mice.^10^ ERK signals were involved in the observed oxytocin‐mediated improvements in learning and memory deficits induced by uncontrollable stress.^29^ Thus, we propose that oxytocin could reverse the Aβ_25–35_‐induced spatial memory impairments via ERK signals and Ca^2+^‐permeable AMPA receptors.

In the current study, we employed the ICV administration of Aβ_25–35_ to induce memory impairments and generate an animal model of AD, according to Maurice et al^25^, ^26^ with a modification such as the addition of AlCl_3_. AlCl_3_ reportedly facilitates the aggregation of β‐amyloid protein^30^ and increases both the reproducibility and the reliability of the induced memory impairments.^2^, ^3^ The amount of AlCl_3_ was estimated based on previous^2^, ^30^ and our preliminary studies. We, herein, demonstrated that the ICV administration of Aβ_25–35_ in mice exerted impairments of spatial working memory as demonstrated by the undertaken Y‐maze test and spatial reference memory as demonstrated by the undertaken MWM test. These results were consistent with previous studies using the Y‐maze and the MWM tests.^31^

As described above, we concluded that oxytocin could become a clinical treatment tool for amnesia in pathologies such as AD. However, the development of peptides as clinical therapeutic tools for CNS disorders is restricted by their limited ability to cross the BBB following a systemic administration.^12^ Since it is very impractical to attempt applying the ICV administration to clinical treatment, we have recently developed a new technique for delivering peptide drugs to the brain via the IN route (European patent: EP‐3‐190‐129‐B1; January 8, 2020). By adding a specific amino acid sequence of PAS and the CPPs to oxytocin, we demonstrated that the resulting oxytocin derivative could be delivered into the brain by IN administration, exerting memory‐improving effects similar to those identified in our previous studies with derivatives of neuropeptides, such as GLP‐2 and neuromedin U.^14^, ^15^ Regarding the mechanisms of the nose‐to‐brain delivery of these derivatives, we recently reported that CPPs enhanced cellular uptake through macropinocytosis. The PAS promoted an escape from the endosomal vesicles and each cell. These properties allow the PAS‐CPPs‐peptide derivatives to “travel” from the nasal mucosa to the neurons inside the brain.^23^ In the present study, dense fluorescence signals observed significantly in the HIP and PVH may indicate that the receptor‐bound oxytocin derivative remained and was detected after the preparation of brain sections, since oxytocin receptors were shown to be abundantly located in the HIP and PVH.^6^, ^8^

Based on these findings, we suggest that oxytocin can positively affect the Aβ_25–35_‐induced impairments of mice's spatial working and spatial reference memory. Furthermore, the oxytocin derivative can be efficiently delivered from the nose to the brain and improve murine memory. We propose that the oxytocin derivative could be useful in the clinical treatment of AD. Further studies are required to clarify a more detailed mechanism for the memory‐related pharmacological effects of oxytocin.

## AUTHOR CONTRIBUTIONS

Junpei Takahashia and Yudai Ueta performed the experiments; Daisuke Yamada, Sachie Sasaki‐Hamada, Takashi Iwaia, and Tomomi Akita conducted the experiments; Chikamasa Yamashita and Akiyoshi Saitoh supported the study and drafted the manuscript, and Jun‐Ichiro Oka designed and supported the study and revised the manuscript. All authors reviewed the manuscript.

## FUNDING INFORMATION

This study was partially supported by JSPS KAKENHI (Grant number 15K07974 to J‐I.O.); the Mochida Memorial Foundation for Medical and Pharmaceutical Research (2015 to S.S‐H.); and the MEXT‐Supported Program for the Strategic Research Foundation at Private Universities (2014–2018 to J‐I.O.). This study was partially supported by a Grant‐in‐Aid for JSPS Fellows (Grant number JP 21J20036 to J.T.).

## CONFLICT OF INTEREST

The authors declare no conflicts of interest.

## INFORMED CONSENT

Not applicable.

## REGISTRY AND THE REGISTRATION NO. OF THE STUDY/TRIAL

Not applicable.

## ANIMAL STUDIES

The Institutional Animal Care and Use Committee at Tokyo University of Science approved all animal study protocols.

## Supporting information


Table S1

Table S2

Table S3

Table S4

Table S5

## Data Availability

The data that support the findings of this study are available in the Supporting Information.
